# P-2015. Evaluating the use of Monoclonal Antibodies - Sotrovimab, Casirivimab/Imedvimab (REGEN-COV) and Tixagevimab/Cilgavimab (EVUSHELD) for COVID-19 Treatment in Singapore

**DOI:** 10.1093/ofid/ofae631.2172

**Published:** 2025-01-29

**Authors:** Annabel Chua, Puay Hoon Eve Nah, Grace Hoo, Ying Hao, Jun Xin Tay, Barnaby Edward Young, Matthias Paul Toh, Mark Chen, Shawn Vasoo

**Affiliations:** Tan Tock Seng Hospital, National Centre for Infectious Diseases, Singapore, Singapore; National Public Health & Epidemiology Unit (NPHEU), National Centre for Infectious Diseases, Singapore, Not Applicable, Singapore; Tan Tock Seng Hospital, National Centre for Infectious Diseases, Singapore, Singapore; National Public Health & Epidemiology Unit (NPHEU), National Centre for Infectious Diseases, Singapore, Not Applicable, Singapore; Tan Tock Seng Hospital, National Centre for Infectious Diseases, Singapore, Singapore; National Centre for Infectious Diseases, Tan Tock Seng Hospital, - National Centre for Infectious Diseases and Tan Tock Seng Hospital, Lee Kong Chian School of Medicine, Singapore, Not Applicable, Singapore; National Public Health & Epidemiology Unit (NPHEU), National Centre for Infectious Diseases, Saw Swee Hock School of Public Health, National University of Singapore, Singapore, Not Applicable, Singapore; National Public Health & Epidemiology Unit (NPHEU), National Centre for Infectious Diseases, Singapore, Not Applicable, Singapore; National Centre for Infectious Diseases and Tan Tock Seng Hospital, Dept of Infectious Diseases, Singapore, Singapore

## Abstract

**Background:**

The emergence of SARS-CoV-2 led to the development of therapeutic monoclonal antibodies (mAbs) including sotrovimab, casirivimab/imdevimab (REGEN-COV) and tixagevimab/cilgavimab (EVUSHELD). While tixagevimab/cilgavimab was not approved in the United States for COVID-19 treatment, there was limited off-label use in Singapore based on promising results from the TACKLE trial. The effectiveness of these mAbs against Omicron variants is uncertain.

**Table 1:**
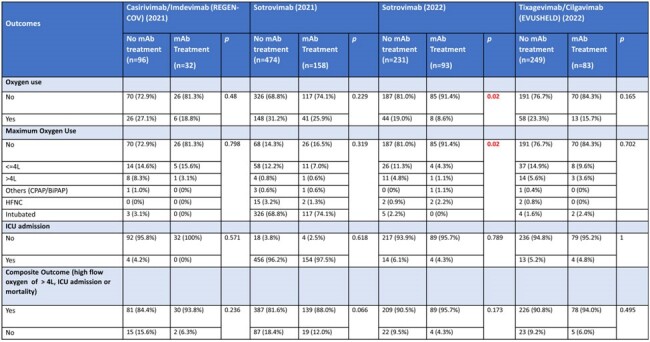
Comparison of outcomes between patients treated with monoclonal antibody and those who did not receive monoclonal antibody treatment

**Methods:**

This is a retrospective cohort study of hospitalized COVID-19 patients treated with mAbs from August 2021 to July 2022, who were matched with a control group at a ratio of 1:3 using propensity score matching (PSM). Characteristics used in PSM included age, gender, vaccination status, comorbidities and use of other treatment modalities ±7 days of 1st PCR positive. Firth’s Logistic regression was used to estimate the effectiveness of mAb in reducing severe COVID-19 outcomes. Patients were stratified by the years 2021 and 2022 to distinguish the Delta and Omicron variants.

**Table 2:**
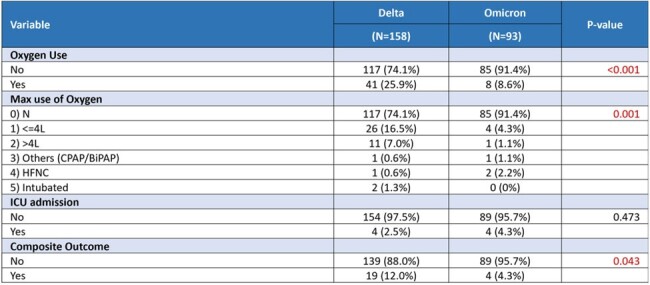
Comparison of outcomes between patient receiving Sotrovimab in 2021 (Delta) and 2022 (Omicron)

**Results:**

A total of 366 patients were treated with mAbs: 251 patients received sotrovimab, 83 tixagevimab/cilgavimab, and 32 casirivimab/imdevimab. Our analysis suggested an overall lower rate of severe COVD-19 outcome including oxygen use and composite outcomes of high flow oxygen ( >4L), Intensive Care Unit (ICU) admission, or mortality when treated with mAbs (Table 1). However, these differences were not statistically significant, except for a lower oxygen use in patients treated with sotrovimab in 2022. Among 93 patients treated with sotrovimab in 2022, 91.4% did not require oxygen use compared to 81.0% not on sotrovimab (p=0.02). When comparing sotrovimab use for Omicron variant in 2022 with the Delta variant in 2021 (n=158), a lower rate of oxygen use (p< 0.001) and composite outcomes (p=0.043) were observed (Table 2). Analysis between sotrovimab and tixagevimab/cilgavimab showed no significant difference in overall effectiveness.

**Table 3:**
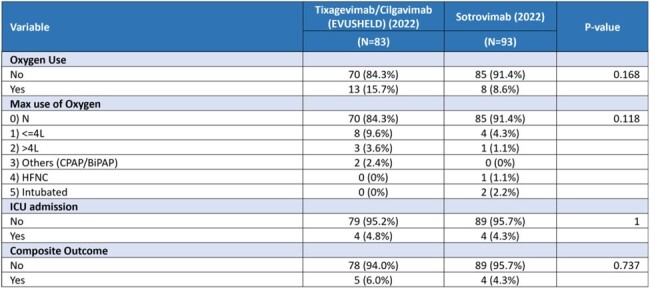
Comparison of outcomes between patients who received Sotrovimab or Tixagevimab/Cilgavimab (EVUSHELD) in 2022

**Conclusion:**

Despite reduced *in vitro* neutralization of Omicron subvariants, there was evidence of improved outcomes among patients who received Sotrovimab. Given the potential for the re-emergence of susceptibility as SARS-CoV-2 continues to evolve, mAbs may continue to serve as a valuable component of the armamentarium.

**Disclosures:**

Barnaby Edward Young, MB BChir, PhD, Astra Zeneca: Honoraria|Gilead: Honoraria|Moderna: Honoraria|Pfizer: Honoraria|Sanofi: Grant/Research Support|Sanofi: Honoraria Shawn Vasoo, MBBS, MRCP, D(ABP), D(ABIM) (Inf Dis), FRCPath, bioMerieux: In-kind, for this study|Rosco Diagnostica: In-kind, for this study

